# The Transient Receptor Potential Melastatin 7 (TRPM7) Inhibitors Suppress Seizure-Induced Neuron Death by Inhibiting Zinc Neurotoxicity

**DOI:** 10.3390/ijms21217897

**Published:** 2020-10-24

**Authors:** Jeong Hyun Jeong, Song Hee Lee, A Ra Kho, Dae Ki Hong, Dong Hyeon Kang, Beom Seok Kang, Min Kyu Park, Bo Young Choi, Hui Chul Choi, Man-Sup Lim, Sang Won Suh

**Affiliations:** 1 Department of Physiology, Hallym University, College of Medicine, Chuncheon 24252, Korea; jd1422@hanmail.net (J.H.J.); sshlee@hallym.ac.kr (S.H.L.); arakho136@naver.com (A.R.K.); zxnm01220@gmail.com (D.K.H.); ehdgus6312@gmail.com (D.H.K.); ttiger1993@gmail.com (B.S.K.); bagmingyu50@gmail.com (M.K.P.); 2 Department of Neurology, Hallym University, College of Medicine, Chuncheon 24252, Korea; 3 Department of Medical Education, Hallym University, College of Medicine, Chuncheon 24252, Korea

**Keywords:** epilepsy, pilocarpine, carvacrol, 2-APB, transient receptor potential melastatin 7, zinc, neuron death, reactive oxygen species

## Abstract

Transient receptor potential melastatin 7 (TRPM7) is an ion channel that mediates monovalent cations out of cells, as well as the entry of divalent cations, such as zinc, magnesium, and calcium, into the cell. It has been reported that inhibitors of TRPM7 are neuroprotective in various neurological diseases. Previous studies in our lab suggested that seizure-induced neuronal death may be caused by the excessive release of vesicular zinc and the subsequent accumulation of zinc in the neurons. However, no studies have evaluated the effects of carvacrol and 2-aminoethoxydiphenyl borate (2-APB), both inhibitors of TRPM7, on the accumulation of intracellular zinc in dying neurons following seizure. Here, we investigated the therapeutic efficacy of carvacrol and 2-APB against pilocarpine-induced seizure. Carvacrol (50 mg/kg) was injected once per day for 3 or 7 days after seizure. 2-APB (2 mg/kg) was also injected once per day for 3 days after seizure. We found that inhibitors of TRPM7 reduced seizure-induced TRPM7 overexpression, intracellular zinc accumulation, and reactive oxygen species production. Moreover, there was a suppression of oxidative stress, glial activation, and the blood–brain barrier breakdown. In addition, inhibitors of TRPM7 remarkably decreased apoptotic neuron death following seizure. Taken together, the present study demonstrates that TRPM7-mediated zinc translocation is involved in neuron death after seizure. The present study suggests that inhibitors of TRPM7 may have high therapeutic potential to reduce seizure-induced neuron death.

## 1. Introduction

Epilepsy is a neurological disorder characterized by unpredictable seizure behavior [1,2,3]. Epileptic seizures are symptoms of abnormally excessive or synchronous neuronal activity in the brain [2]. The most common form of epilepsy, which does not occur congenitally, is temporal lobe epilepsy (TLE), which is one of the most common and deadly neurological disorders and one of the leading causes of cognitive impairment [4]. Epileptic seizures can also be caused by various types of brain damage, such as hypoxia, head trauma, or multiple recessive seizures [5,6]. Seizure activity may be disseminated through the brain’s endogenous electrical fields. The anticipated mechanisms that may diffuse the spread and recruitment of neurons produce an increase in K^+^ concentration outside of the cell and an increase in Ca^2+^ in the presynaptic terminals [7]. These mechanisms may blunt hyperpolarization and depolarize proximate neurons, as well as increase neurotransmitter release. Another mechanism of epilepsy that occurs after brain injury is an imbalance between neuronal excitation and inhibition, such as the up-regulation of excitatory circuits or the down-regulation of inhibitory circuits [8,9]. Thus, persistent and uncontrolled epilepsy can cause permanent brain damage and result in a cognitive decline [3]. In particular, epileptic seizures have a severe deteriorating effect on the function of the hippocampus, causing neuronal damage and gradually accumulating harmful cellular and metabolic changes [10,11]. In addition, epilepsy has been shown to increase spontaneous seizures by altering the development of the hippocampus and synapse formation [12]. Nevertheless, many survivors of severe epileptic seizures still show neuronal damage and cognitive impairment, while the rate of cure is significantly lower [13,14].

Zinc is the second most abundant transition metal after iron in the central nervous system [15,16]. Zinc modulates various physiological functions, such as cell division, proliferation, migration, development, and DNA synthesis [17,18]. Zinc deficiency can degrade the performance of short-term memory operations. Conversely, too much zinc is known to drive increased neuronal death after brain injury. Therefore, it is important to maintain the homeostasis of zinc under physiological conditions [19,20,21]. Most zinc exists in association with zinc-binding protein. However, in cases of neurological damage, excessive zinc is released into the extracellular space from the presynaptic terminals of neurons and then translocates into the intracellular space of postsynaptic neurons [20,22]. This leads to increased levels of intracellular free zinc, which can produce neurotoxicity [21]. Previous studies have consistently demonstrated that the influx of excess zinc into neurons following neurological disease, such as epilepsy [19,23,24,25,26], ischemia [27], trauma [28], and hypoglycemia [29,30], induces neuronal death.

The transient receptor potential melastatin 7 (TRPM7), belonging to the subfamily of the TRP channel, is an ion channel that regulates monovalent cations out of cells and the entry of divalent cations, such as zinc, magnesium, and calcium, into the cell [31]. In addition, TRPM7 also mediates the movement of divalent cations out of cells, as well as the entry of monovalent cations. In such cases, the extracellular divalent cation concentration is much higher than the intracellular concentration, but K^+^ can escape via TRPM7 [32]. TRPM7 is the only known zinc permeable channel among the TRP family of ion channels [33,34]. In addition, the TRPM7 channel offers zinc permeability that is four-fold higher than calcium [33]. Previous studies have shown that the knockdown of TRPM7 with small interfering RNA and nonspecific TRPM7 inhibitors, such as Gd^3+^ or 2-aminoethoxydiphenyl borate (2-APB), decrease zinc neurotoxicity in cultured mouse cortical neurons [35].

Carvacrol, which is an essential oil extracted from *Origanum vulgare* (oregano), is well known to effectively act as an inhibitor of TRPM7 [36,37]. Carvacrol, a monoterpenoid phenol, is not a specific inhibitor of TRPM7 and may have other effects dependent on binding to other TRPC channels [38,39]. Carvacrol treatment reduced recurrent status epilepticus (SE)-induced cell death in cornu Ammonis 1 (CA1) and hilus [40], possibly through its action on TRPM7 channels. A recent study demonstrated that carvacrol inhibits lipopolysaccharide (LPS)-induced pro-inflammatory activation in RAW 264.7 macrophages via the ERK1/2 and NF-kB pathways [41]. In addition, there are recent reports that carvacrol has a neuroprotective effect by reducing TRPM7 overexpression and caspase3 activity in a hemiparkinsonian model [42]. Moreover, there are recent reports that essential extracts such as carvacrol have anticonvulsant effects [43,44]. In addition, it has been reported that carvacrol has a neuroprotective effect on central nervous system (CNS) diseases, such as ischemia, and traumatic brain injury (TBI) [45,46,47,48]. To further confirm the conclusion, we determined whether 2-aminoethoxydiphenyl borate (2-APB), another non-specific inhibitor of TRPM7, promoted neuroprotective effects after seizure with a pilocarpine-induced SE model [49]. 2-APB is a membrane permeable lipophilic compound that acts as a blocker of the intracellular inositol 1,4,5-trisphosphate receptor (IP_3_R) [50,51]. Although 2-APB is not specific, it is also known to act as an inhibitor of TRPM7 [52,53]. A recent study demonstrated that 2-APB has neuroprotective effects after ischemia by inhibiting the TRPM7 channel [54].

Therefore, the present study tested our hypothesis that TRPM7 channel inhibition may prevent zinc translocation and subsequent neuronal death after epilepsy. To evaluate our hypothesis, we used two different TRPM7 inhibitors, carvacrol and 2-APB, using lithium-pilocarpine-induced status epilepticus (SE) as a model for TLE.

## 2. Results

### 2.1. Carvacrol Reduces Seizure-Induced TRPM7 Overexpression, Zinc Accumulation, and Neuronal Death

**Figure 1:**
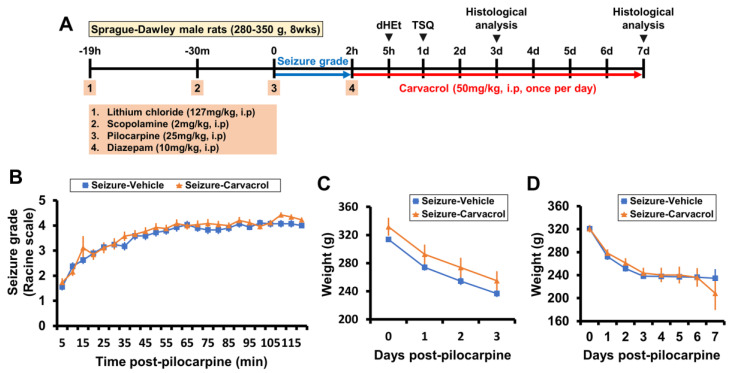
Carvacrol did not affect body weight following pilocarpine-induced status epilepticus (SE). (**A**) Timeline showing the experimental procedures. SE was induced by lithium-pilocarpine injection. Carvacrol was intraperitoneally administered once per day for all experimental periods. (**B**) Line graphs showing the seizure grade based on the Racine stage during SE induction by pilocarpine (mean ± SEM; *n* = 26–29 per group). (**C**,**D**) Graphs representing the weight change for 3 (**C**) or 7 days (**D**) after SE (mean ± SEM; *n* = 8–10 per group).

To test whether blocking the TRPM7 channel by carvacrol could reduce the accumulation of intracellular free zinc and thereby rescue neurons from delayed neuronal death, we used a lithium-pilocarpine-induced SE model. Carvacrol was injected intraperitoneally once per day at a dose of 50 mg/kg for the entire experimental period following pilocarpine-induced SE. The brains were harvested at the designated time after SE for histological evaluation (Figure 1A). We first determined seizure behaviors according to the Racine scale [55] and used only SE-induced rats in this experiment (Figure 1B). Furthermore, we assessed weight change as a possible side effect after application of carvacrol. However, carvacrol treatment did not result in significant side effects in terms of weight loss (Figure 1C,D).

**Figure 2:**
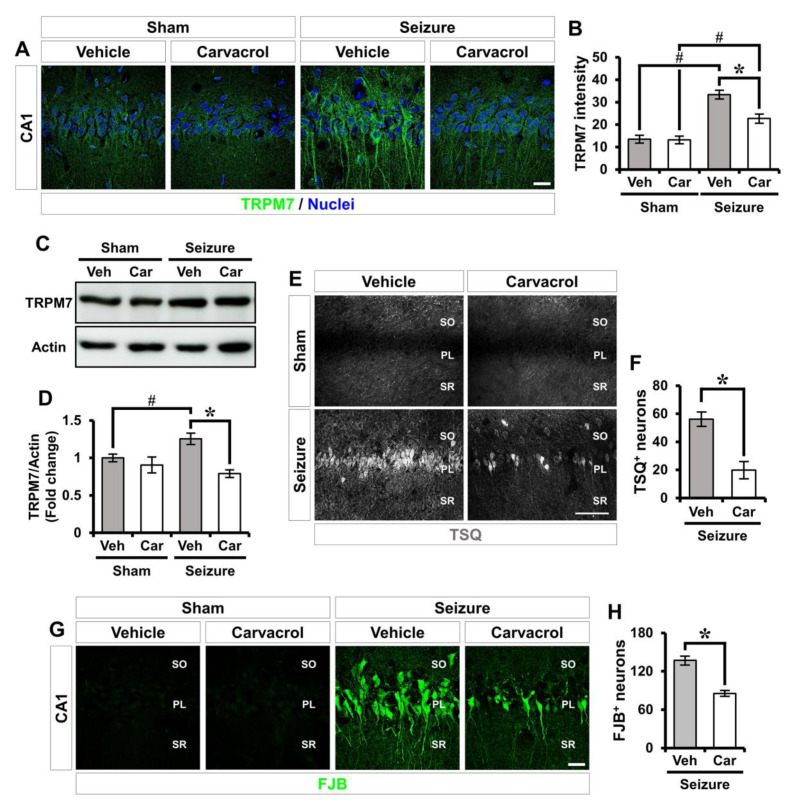
Carvacrol treatment reduces TRPM7 overexpression, zinc accumulation, and neuronal degeneration after seizure. (**A**) Representative images showing TRPM7 immunoreactivity (green) in the CA1 of the hippocampus. Nuclei are counterstained with DAPI (blue). Scale bar = 20 µm. (**B**) The bar graph representing the immunofluorescence intensity of TRPM7 as determined in the same hippocampal region (mean ± SEM; *n* = 5 from each sham group, *n* = 7 from each seizure group). * *p* < 0.05 vs. the vehicle-treated group; # *p* < 0.05 vs. the sham-operated group (Kruskal–Wallis test followed by a Bonferroni post-hoc test: chi square = 19.452, df = 3, *p* < 0.001). (**C**) Western blot analysis of TRPM7 in the hippocampus. (**D**) Quantification of TRPM7 protein levels from the hippocampus. (mean ± SEM; *n* = 5 from each sham group, *n* = 7–8 from each seizure group). * *p* < 0.05 vs. the vehicle-treated group; # *p* < 0.05 vs. the sham-operated group (Kruskal–Wallis test followed by a Bonferroni post-hoc test: chi square = 12.310, df = 3, *p* < 0.006). (**E**) Representative images showing sections of the hippocampus stained with TSQ to detect zinc accumulation. Scale bar = 100 µm. (**F**) Bar graph showing the number of TSQ^+^ neurons in the hippocampal CA1 (mean ± SEM; *n* = 6 from each sham group, *n* = 5 from each seizure group). * *p* < 0.05 vs. the vehicle-treated group (Mann–Whitney U test: *z* = 2.611, *p* = 0.008). (**G**) Fluorescent images representing the degenerating neurons stained with Fluoro-Jade B (FJB) in the hippocampal CA1. Scale bar = 20 µm. (**H**) Quantification of the number of FJB^+^ neurons (mean ± SEM; *n* = 5 from each sham group, *n* = 8 from each seizure group). * *p* < 0.05 vs. the vehicle-treated group (Mann–Whitney U test: *z* = 1.785, *p* = 0.074).

To investigate whether carvacrol could inhibit the TRPM7 channel after seizure, we used immunofluorescence staining to analyze the expression of TRPM7. Like previous studies, we found that TRPM7 exists primarily on cell bodies and is processed in hippocampal CA1 pyramidal neurons. In the sham-operated groups, there was no significant difference in TRPM7 expression between the vehicle- and carvacrol-treated groups. The vehicle-treated seizure group revealed a significant up-regulation of TRPM7 expression at 3 days following pilocarpine-induced SE. However, carvacrol treatment remarkably reduced TRPM7 expression after SE (sham-vehicle, 13.5 ± 1.6; sham-carvacrol, 13.2 ± 1.5, seizure-vehicle, 32.6 ± 1.7; seizure-carvacrol, 22.7 ± 1.8, a 30.4% decrease; Figure 2A,B). Western blot also revealed a significant increase in the level of TRPM7 protein in the hippocampus 24 h after pilocarpine-induced SE as compared with the sham-operated groups. However, administration of carvacrol significantly decreased the protein level of TRPM7, compared to the vehicle-treated SE group (sham-vehicle, 1 ± 0.07; sham-carvacrol, 0.994 ± 0.08, seizure-vehicle, 1.412 ± 0.15; seizure-carvacrol, 0.965 ± 0.07, a 31.7% decrease; Figure 2C,D). Next, to examine the effects of carvacrol on seizure-induced zinc accumulation and neuronal degeneration, we performed TSQ (6-methoxy-8-p-toluenesulfonamido-quinoline) and Fluoro Jade-B (FJB) staining on the brains harvested 24 h and 3 days after SE, respectively. TSQ, which can bind free or loosely bound zinc, is a commonly used zinc sensor [56,57,58], and FJB is useful for the detection of degenerating neurons [59]. SE induced by lithium-pilocarpine led to considerable accumulation of intracellular free zinc and neuronal death in hippocampal CA1. Compared to the vehicle-treated group, the number of TSQ^+^ neurons was remarkably decreased in the carvacrol-treated group (seizure-vehicle, 56 ± 5.6; seizure-carvacrol, 20 ± 6.6, a 64.3% decrease; Figure 2E,F). In addition, FJB staining also showed that carvacrol treatment significantly reduced the number of degenerating neurons (seizure-vehicle, 197 ± 26.5; seizure-carvacrol, 117 ± 20, a 40.6% decrease; Figure 2G,H). These findings indicate that carvacrol treatment reduces the accumulation of intracellular free zinc by inhibiting the overexpression of the TRPM7 channel, which occurs in the hippocampal CA1 following pilocarpine-induced SE, thereby causing a reduction in neuronal degeneration.

### 2.2. Carvacrol Reduces Seizure-Induced Superoxide Production and Oxidative Stress

**Figure 3:**
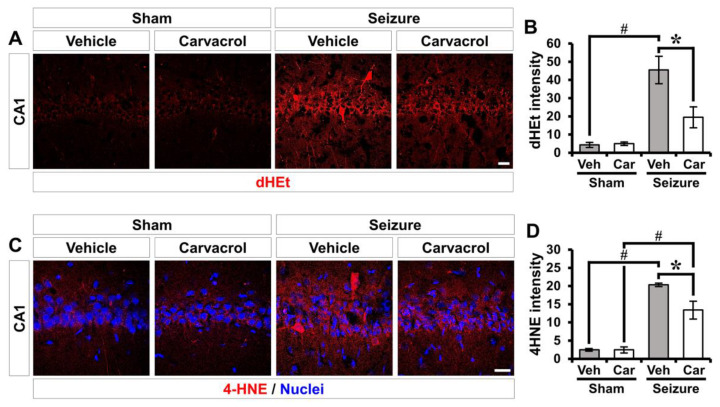
Carvacrol treatment reduces reactive oxygen species (ROS) production and oxidative stress after seizure. (**A**) Representative images showing the Et fluorescence in the hippocampal CA1. Scale bar = 20 µm. (**B**) The bar graph representing the intensity of Et fluorescence as determined in the same hippocampal region (mean ± SEM; *n* = 6 from each sham group, *n* = 5–6 from each seizure group). * *p* < 0.05 vs. the vehicle-treated group (Kruskal–Wallis test followed by a Bonferroni post-hoc test: chi square = 16.579, df = 3, *p* < 0.001). (**C**) Immunofluorescent images representing the lipid peroxidation stained for anti-4HNE (red) in the hippocampal CA1. Nuclei are counterstained with DAPI (blue). Scale bar = 20 µm. (**D**) Quantification of the immunofluorescence intensity of 4HNE as determined in the same hippocampal region (mean ± SEM; *n* = 5 from each sham group, *n* = 7 from each seizure group). * *p* < 0.05 vs. the vehicle-treated group; # *p* < 0.05 vs. the sham-operated group (Kruskal–Wallis test followed by a Bonferroni post-hoc test: chi square = 19.853, df = 3, *p* < 0.001).

To determine whether carvacrol could affect reactive oxygen species (ROS) production and oxidative stress in the hippocampal CA1 following pilocarpine-induced SE, we analyzed Et fluorescence and immunofluorescence by dihydroethidium (dHEt) and 4-hydroxynonenal (4HNE) staining, respectively. dHEt and 4HNE are used as indicators of ROS or lipid peroxidation, respectively. SE induced by lithium-pilocarpine gave rise to increased Et fluorescence and 4HNE intensity in the hippocampal CA1. Carvacrol treatment remarkably reduced the intensity of the Et signal and 4HNE immunofluorescence after SE compared to the vehicle-treated SE group (Figure 3A,B: sham-vehicle, 4.3 ± 1.7; sham-carvacrol, 5 ± 1.3, seizure-vehicle, 45.5 ± 7.9; seizure-carvacrol, 19.5 ± 6.1, a 57.1% decrease; Figure 3C,D: sham-vehicle, 2.6 ± 0.6; sham-carvacrol, 1.8 ± 0.2, seizure-vehicle, 21.6 ± 1.2; seizure-carvacrol, 13.5 ± 1.9, a 37.5% decrease).

### 2.3. Carvacrol Reduces Glial Activation after Pilocarpine-Induced Seizure

**Figure 4:**
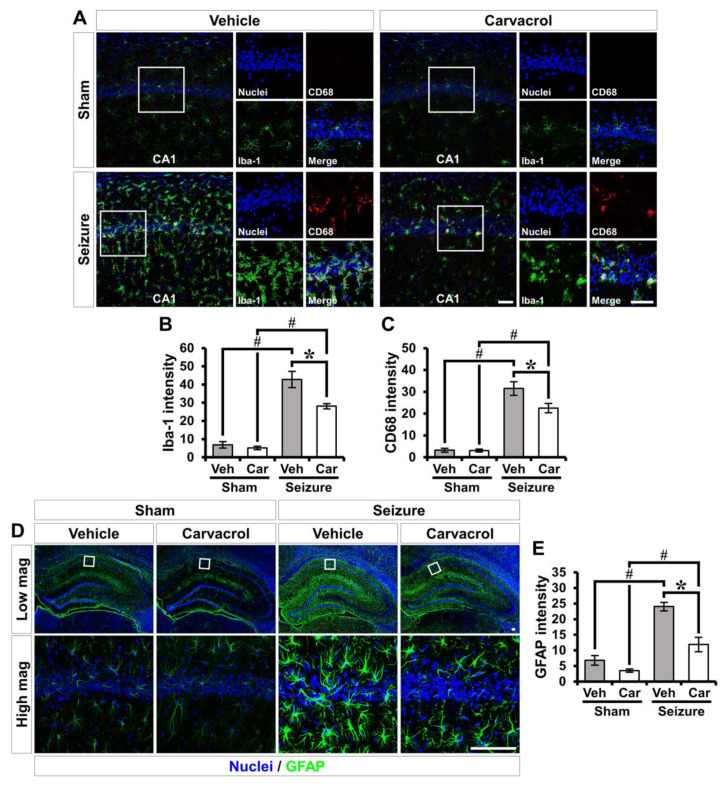
Carvacrol treatment reduces seizure-induced glial activation. (**A**) Double-label confocal micrographs of Iba-1 (green) and CD68 (red) in the hippocampal CA1 from the vehicle- and carvacrol-treated groups after sham or SE. Nuclei stained with DAPI (blue). Scale bar, 50 µm. (**B**,**C**) Bar graphs showing the intensity of Iba-1 (**B**) and CD68 (**C**) as determined in the same hippocampal region (mean ± SEM; *n* = 5 from each sham group, *n* = 6–8 from each seizure group). * *p* < 0.05 vs. the vehicle-treated group; # *p* < 0.05 vs. the sham-operated group (Kruskal–Wallis test followed by a Bonferroni post-hoc test; Figure 4B: chi square = 19.244, df = 3, *p* < 0.001; Figure 4C: chi square = 18.708, df = 3, *p* < 0.001). (**D**) Immunofluorescent images representing the astrocyte activation stained for anti-GFAP (green) in the hippocampus. Nuclei are counterstained with DAPI (blue). Scale bar = 100 µm. (**E**) Quantification of the immunofluorescence intensity of GFAP as determined in the same hippocampal region (mean ± SEM; *n* = 4–5 from each sham group, *n* = 7 from each seizure group). * *p* < 0.05 vs. the vehicle-treated group; # *p* < 0.05 vs. the sham-operated group (Kruskal–Wallis test followed by a Bonferroni post-hoc test: chi square = 16.32, df = 3, *p* = 0.001).

We next assessed the effects of carvacrol treatment on microglia/macrophage activation following pilocarpine-induced SE. The activation of microglia and macrophages was monitored by double immunofluorescence staining using antibodies against the ionized calcium-binding adapter molecule 1 (Iba-1) and the cluster of differentiation 68 (CD68). Iba-1 is constitutively expressed in microglia/macrophages, and CD68 is a lysosome-associated membrane protein. Colocalization with Iba-1 and CD68 is well known as an indicator of microglia/macrophage M1 polarization [60]. The sham-operated groups showed ramified microglia/macrophage morphology in a resting state. The Iba-1 immunoreactivity in the vehicle-treated sham group was similar to that in the carvacrol-treated sham group. The vehicle-treated SE group had prominent microglia/macrophage activation in the hippocampal CA1. In addition, many Iba-1^+^ microglia/macrophages were significantly co-localized with CD68 at 3 days following pilocarpine-induced SE. In contrast, carvacrol treatment decreased the intensity of Iba-1 and CD68 immunofluorescence after SE (sham-vehicle, 6.9 ± 1.5; sham-carvacrol, 5.1 ± 0.6, seizure-vehicle, 42.8 ± 4.7; seizure-carvacrol, 28.1 ± 1.2, a 34.3% decrease, sham-vehicle, 3.3 ± 0.6; sham-carvacrol, 3.1 ± 0.4, seizure-vehicle, 31.5 ± 3.3; seizure-carvacrol, 22.5 ± 2.3, a 28.6% decrease; Figure 4A–C). We also investigated whether carvacrol could decrease astroglial activation following pilocarpine-induced SE. The activation of astrocyte was monitored by immunofluorescence staining using an antibody against the astrocyte-specific marker glial fibrillary acidic protein (GFAP). Three days after SE, the GFAP^+^ astrocyte in the vehicle-treated SE group clearly showed hypertrophied processes, which indicates the development of astrogliosis and the activation of astrocytes. However, carvacrol treatment remarkably decreased astrogilal activation in the hippocampus after SE (sham-vehicle, 7.4 ± 1.4; sham-carvacrol, 3.7 ± 0.3, seizure-vehicle, 24.1 ± 1.5; seizure-carvacrol, 11.9 ± 2.5, a 50.6% decrease; Figure 4D,E).

### 2.4. Carvacrol Reduces Seizure-Induced BBB Breakdown and Vessel Disorganization

**Figure 5:**
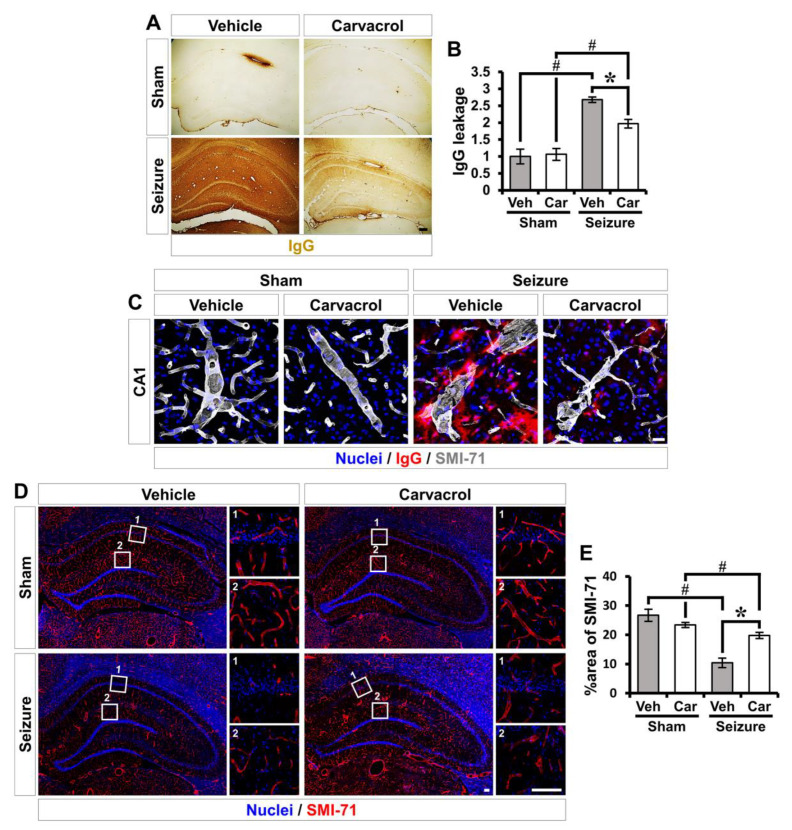
Carvacrol treatment reduces the blood–brain barrier (BBB) disruption and vessel disappearance following pilocarpine-induced SE. (**A**) Photomicrographs showing sections of the hippocampus stained for anti-mouse immunoglobulin G (IgG) to detect endogenous IgG. Scale bar, 100 µm. (**B**) Graph representing IgG leakage from the hippocampus in mice treated with the vehicle or carvacrol at 3 days following pilocarpine-induced SE (mean ± SEM; *n* = 5 from each sham group, *n* = 7 from each seizure group). * *p* < 0.05 vs. the vehicle-treated group; # *p* < 0.05 vs. the sham-operated group (Kruskal–Wallis test followed by Bonferroni post-hoc test; chi square = 18.493, df = 3, *p* < 0.001). (**C**) Double Immunofluorescent images representing the BBB marker SMI-71^+^ endothelial protein (gray) and endogenous IgG leakage (red) in the hippocampus. Nuclei are counterstained with DAPI (blue). Scale bar = 20 µm. (**D**) Representative images showing the SMI-71^+^ endothelial protein (red) in the hippocampus. Nuclei are counterstained with DAPI (blue). Scale bar, 100 µm. (**E**) Quantification of the percent area of the SMI-71^+^ endothelial protein in the hippocampus (mean ± SEM; *n* = 3–4 from each sham group, *n* = 5–7 from each seizure group). * *p* < 0.05 vs. the vehicle-treated group; # *p* < 0.05 vs. the sham-operated group (Kruskal–Wallis test followed by a Bonferroni post-hoc test: chi square = 19.080, df = 3, *p* < 0.001).

To analyze the putative blood–brain barrier (BBB) damage, we sought to confirm the leakage of serum IgGs using immunohistochemistry. In the sham-operated groups, there was little or no leakage of the IgGs, whereas, in the pilocarpine-induced SE groups, we observed a significant increase in IgG immunoreactivity throughout the hippocampus. Compared to the vehicle-treated SE group, IgG immunoreactivity was significantly reduced in in the hippocampus of the carvacrol-treated SE group (sham-vehicle, 1 ± 0.2; sham-carvacrol, 1.1 ± 0.2, seizure-vehicle, 2.7 ± 0.1; seizure-carvacrol, 2.0 ± 0.1, a 25.9% decrease; Figure 5A,B). We also checked the extravasation of endogenous serum IgGs from vessels following pilocarpine-induced SE. Sections were stained for endogenous IgG and the endothelial protein found in areas with BBB (SMI-71), which can be used as an indicator of BBB damage, to highlight vascular permeability. The vehicle-treated SE group showed pronounced and diffuse IgG immunoreactivity in the vessels of the hippocampus, reflecting endogenous serum protein extravasation, which obscured the boundaries between the vessel segments. However, carvacrol treatment significantly decreased IgG immunoreactivity in the vessels, which suggests that the carvacrol preserved the integrity of the BBB (Figure 5C). In addition, we examined the distribution of SMI-71^+^ blood vessels in the hippocampus at 3 days post SE. The SMI-71^+^ vessels were widely distributed throughout the hippocampus. The vehicle-treated SE group showed a considerable disappearance of SMI-71^+^ blood vessels, particularly in the molecular layer of the dentate gyrus (DG) and the hippocampal CA1 subfield. By contrast, carvacrol treatment significantly inhibited the disappearance of SMI-71^+^ vessels in the hippocampus (sham-vehicle, 26.8 ± 3; sham-carvacrol, 23.5 ± 0.7, seizure-vehicle, 10.8 ± 2.3; seizure-carvacrol, 19.6 ± 0.7, a 81% increase; Figure 5D,E). These results indicate that carvacrol treatment preserved BBB integrity and inhibited vessel disorganization following pilocarpine-induced SE.

### 2.5. Carvacrol Reduces Seizure-Induced Apoptotic Neuronal Death

**Figure 6:**
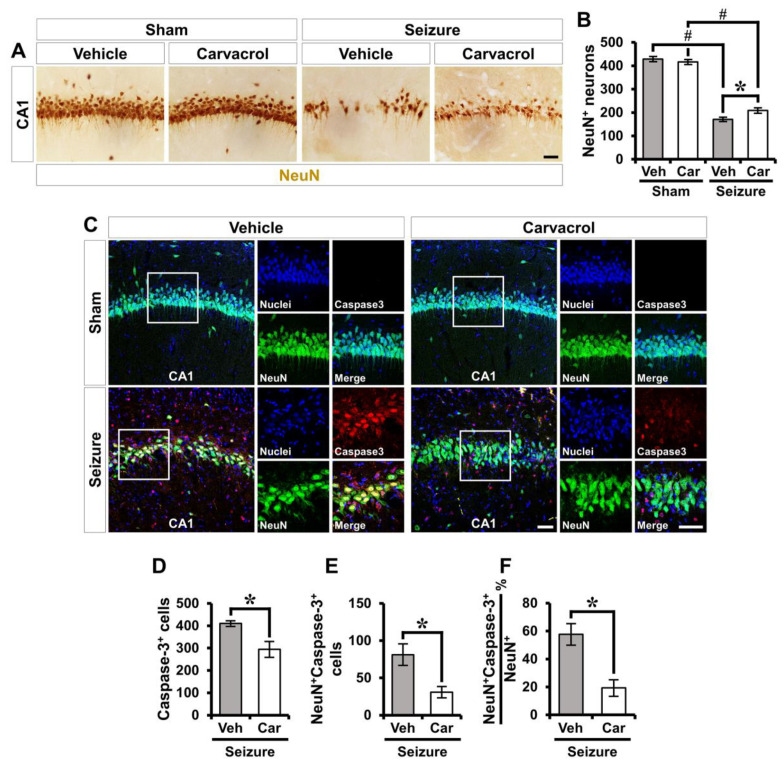
Carvacrol treatment reduces apoptotic neuronal death following pilocarpine-induced SE. (**A**) Representative images showing the expression of NeuN^+^ live neurons in the hippocampal CA1 from the vehicle- and carvacrol-treated groups 1 week after sham or SE. Scale bar, 100 µm. (**B**) Quantification showing the number of NeuN^+^ neurons as determined in the same hippocampal region (mean ± SEM; *n* = 6 from each sham group, *n* = 10 from each seizure group). * *p* < 0.05 vs. the vehicle-treated group; # *p* < 0.05 vs. the sham-operated group (Kruskal–Wallis test followed by a Bonferroni post-hoc test; chi square = 23.958, df = 3, *p* < 0.001). (**C**) Double immunofluorescent images representing the neuronal marker NeuN^+^ cells (green) co-labeled with the cleaved caspase-3 (red) in the hippocampal CA1 from the vehicle- and carvacrol-treated groups after SE. The nuclei are counterstained with DAPI (blue). Scale bar, 50 µm. (**D**–**F**) Quantification of the number of caspase-3^+^ (**D**) and NeuN^+^Caspase-3^+^ cells (**E**) and the percent of NeuN^+^Capase-3^+^ cells over total NeuN^+^ cells (**F**) as determined in the same hippocampal region (mean ± SEM; *n* = 4–6 per group). * *p* < 0.05 vs. the vehicle-treated group (Mann–Whitney U test: Figure 6D: *z* = 2.082, *p* = 0.041; Figure 6E: *z* = 2.242, *p* = 0.026; Figure 6F: *z* = 2.882, *p* = 0.002).

To assess whether carvacrol has neuroprotective effects, we analyzed the delayed neuronal loss at 7 days following pilocarpine-induced SE. The sham-operated groups showed intense staining of the neuronal nuclei (NeuN)^+^ neurons in the hippocampal CA1. There was no significant difference in the number of NeuN^+^ neurons between the vehicle- and carvacrol-treated groups. Pilocarpine-induced SE led to a significant reduction in the number of NeuN^+^ neurons compared to the sham-operated groups. The number of NeuN^+^ neurons was remarkably higher in the carvacrol-treated group than in the vehicle-treated group (sham-vehicle, 429 ± 13.9; sham-carvacrol, 416 ± 11.9, seizure-vehicle, 171 ± 10.5; seizure-carvacrol, 209 ± 13.3, a 22% increase; Figure 6A,B). We next asked how carvacrol protects hippocampal CA1 pyramidal neurons by determining the differences in the apoptotic neuronal death in the vehicle- and carvacrol-treated SE groups. To examine this, we conducted double immunofluorescence staining using antibodies against NeuN and cleaved caspase-3, which is an indicator of cell death during apoptosis. The number of caspase-3^+^ and NeuN^+^caspase-3^+^ cells was significantly reduced in the carvacrol-treated SE group compared to the vehicle-treated SE group. We also found that carvacrol treatment revealed a significant decrease in the percentage of NeuN+Caspase-3+ cells among NeuN^+^ cells after SE compared to the vehicle-treated SE group (seizure-vehicle, 383 ± 22.5; seizure-carvacrol, 262 ± 34.7, a 31.6% decrease, seizure-vehicle, 71 ± 11.8; seizure-carvacrol, 31 ± 8.4, a 56.3% decrease, seizure-vehicle, 55 ± 5.4; seizure-carvacrol, 19 ± 6.3, a 65.5% decrease; Figure 6C–F). These results suggest that carvacrol reduces apoptotic neuronal death following pilocarpine-induced SE.

### 2.6. 2-APB Reduces Seizure-Induced TRPM7 Overexpression, Zinc Accumulation, and Neuronal Death

**Figure 7:**
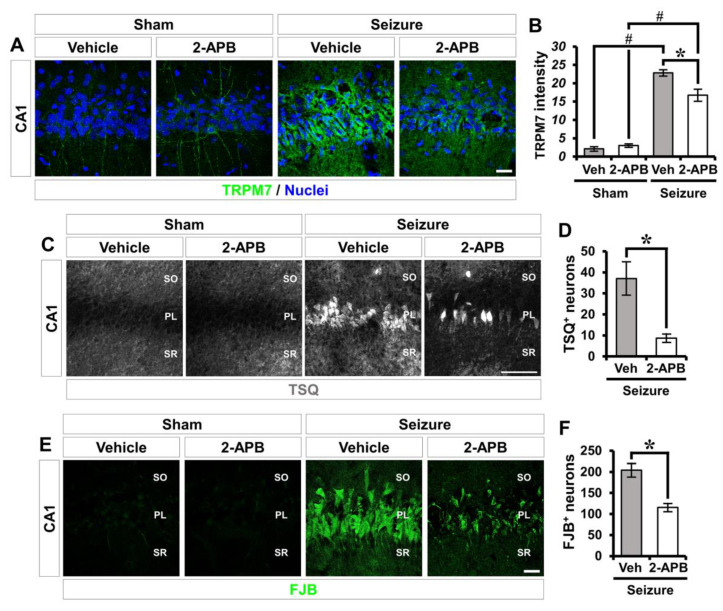
2-APB treatment reduces TRPM7 overexpression, zinc accumulation, and neuronal degeneration after seizure. (**A**) Representative images showing TRPM7 immunoreactivity (green) in the CA1 of the hippocampus. Nuclei are counterstained with DAPI (blue). Scale bar = 20 µm. (**B**) The bar graph representing the immunofluorescence intensity of TRPM7 as determined in the same hippocampal region (mean ± SEM; *n* = 5 from each sham group, *n* = 5–6 from each seizure group). * *p* < 0.05 vs. the vehicle-treated group; # *p* < 0.05 vs. the sham-operated group (Kruskal–Wallis test followed by a Bonferroni post-hoc test: chi square = 17.451, df = 3, *p* < 0.001). (**C**) Representative images showing sections of the hippocampus stained with TSQ to detect zinc accumulation. Scale bar = 100 µm. (**D**) Bar graph showing the number of TSQ^+^ neurons in the hippocampal CA1 (mean ± SEM; *n* = 6 from each sham group, *n* = 5–6 from each seizure group). * *p* < 0.05 vs. the vehicle-treated group (Mann–Whitney U test: *z* = 2.739, *p* = 0.004). (**E**) Fluorescent images representing the degenerating neurons stained with Fluoro-Jade B (FJB) in the hippocampal CA1. Scale bar = 20 µm. (**F**) Quantification of the number of FJB^+^ neurons (mean ± SEM; *n* = 5 from each sham group, *n* = 5–6 from each seizure group). * *p* < 0.05 vs. the vehicle-treated group (Mann–Whitney U test: *z* = 2.373, *p* = 0.017).

To further confirm our initial results, we tested whether 2-APB, another non-specific inhibitor of TRPM7, promoted neuroprotective effects after seizure with a pilocarpine-induced SE model. To investigate whether 2-APB could alter the expression of the TRPM7 channel after seizure, we used immunofluorescence staining to analyze surface expression of TRPM7. In the sham-operated groups, there was no significant difference in TRPM7 expression between the vehicle- and carvacrol-treated groups. The vehicle-treated seizure group revealed a significant up-regulation of TRPM7 expression at 3 days following pilocarpine-induced SE. However, carvacrol treatment remarkably reduced TRPM7 expression after SE (sham-vehicle, 2.1 ± 0.4; sham-carvacrol, 3 ± 0.3, seizure-vehicle, 22.8 ± 1.0; seizure-carvacrol, 16.7 ± 1.8, a 26.8% decrease; Figure 7A,B). To determine whether 2-APB affects zinc accumulation and neuronal death after seizure, we performed TSQ (6-methoxy-8-p-toluenesulfonamido-quinoline) and Fluoro-Jade B (FJB) staining. SE induced by lithium-pilocarpine led to considerable accumulation of intracellular free zinc and neuronal death in hippocampal CA1. Compared to the vehicle-treated group, the number of TSQ^+^ neurons was remarkably decreased in the 2-APB-treated group (seizure-vehicle, 37 ± 8.2; seizure-carvacrol, 9 ± 2.3, a 75.7% decrease; Figure 7C,D). In addition, FJB staining also showed that 2-APB treatment significantly reduced the number of degenerating neurons (seizure-vehicle, 204 ± 17.5; seizure-carvacrol, 115 ± 11.2, a 43.6% decrease; Figure 7E,F). These findings indicate that 2-APB treatment also reduces the accumulation of intracellular free zinc by inhibiting the overexpression of the TRPM7 channel, which occurs in the hippocampal CA1 following pilocarpine-induced SE, thereby causing a reduction in neuronal degeneration.

## 3. Discussion

Using lithium-pilocarpine-induced SE as a model for TLE, we investigated the therapeutic potential of carvacrol to protect against hippocampal neuron death induced by the zinc-mediated pathogenic mechanisms following SE. Here, we found that carvacrol reduced seizure-induced TRPM7 overexpression, intracellular zinc accumulation, and ROS production. Moreover, we observed the suppression of oxidative stress, glial activation, and BBB breakdown. In addition, carvacrol remarkably decreased apoptotic neuron death following seizure. Thus, the neuroprotective effects of carvacrol in pilocarpine-induced SE may occur through the inhibition of TRPM7 and the subsequent reduction in intracellular free zinc accumulation.

Zinc plays an important role in maintaining the immune system, metabolic homeostasis, and antioxidant activity [61]. However, in pathological conditions such as TBI, ischemia, and seizure (which indicate a devastating state of the CNS), synaptically released zinc excessively enters into the intracellular space [62]. The accumulation of intracellular free zinc can induce neuronal death [19,25,28,30].

TRPM7 is a selective cation permeable channel with serine/threonine kinase activity [63]. Divalent cations such as Ca^2+^, Zn^2+^, and Mg^2+^ that enter the cell through the TPRM7 channel play an important role in neuronal apoptosis [64]. However, among the cations that enter into the cell through the TRPM7 channel, zinc is known to have the highest affinity [65]. In addition, activation of the TRPM7 channel increases oxidative stress. Increased oxidative stress stimulates the TRPM7 channels more strongly, leading to divalent cation permeation through these channels [35,66]. A molecular study also showed that the TRPM7 channels affect zinc-mediated neurotoxicity [26,35]. Recently, Doboszewska et al. has suggested that the neuroprotective effects of carvacrol may be related to reduction of intracellular free zinc levels via inhibition of TRPM7 [26]. In the present study, we also hypothesize that inhibiting the TRPM7 channel is expected to inhibit zinc from entering the cell through this channel. Thus, the purpose of this study was to identify the effect of carvacrol on zinc-mediated neuron death after pilocarpine-induced SE. We first assessed TRPM7 expression to determine if carvacrol administration inhibited TRPM7 expression. As a result, we confirmed that overexpression of the TRPM7 channel increased after a seizure and decreased via the administration of carvacrol. Carvacrol may not directly down regulate TRPM7 protein expression but suppress a second round of up-regulation after initial expression. Seizures cause a significant increase in the level of free zinc in the hippocampus of the brain [62]. Among the cations that enter into the TRPM7 channel, zinc is known to have the highest affinity [33]. By inhibiting TRPM7 channels, the level of free zinc entering the cell can be reduced. In this way, it is possible to reduce the excessive accumulation of free zinc into the cell, which will reduce neuronal death. We found that zinc translocation and accumulation was reduced by carvacrol treatment. Because carvacrol is a non-specific inhibitor of the TRPM7 channel, we confirmed the effect of another non-specific inhibitor, 2-APB. 2-APB is known as an inhibitor of the TRP channel and an inhibitor of the IP3 receptor [52,67]. It has been reported that zinc toxicity in mouse cortical neurons was reduced by administration of 2-APB [35]. Therefore, we used 2-APB, another non-specific TRPM7 channel inhibitor, to confirm the protective effect of inhibiting TRPM7 activity on neuronal death after seizure. Administration of 2-APB after seizure reduced TRPM7 channel overexpression, zinc accumulation and neuronal death (Figure 7).

Oxidative injury, glial activation, and BBB disruption are involved in the process leading to neuronal death after seizures [68,69,70]. The excessive production of ROS gives rise to mitochondrial dysfunction and consequently resulted in neuronal death [71,72]. Antioxidant activity decreased after seizure, which also decreased antioxidant activity, thereby making the brain more susceptible to oxidative stress, as indicated by increased lipid peroxidation [73]. Numerous studies have demonstrated that zinc release is an upstream event of ROS production. The excessive activity of the TRPM7 channel and excessive accumulation of zinc also induced reactive oxygen species (ROS) [74,75]. The activated ROS acts to activate the TRPM7 channel and vice versa [74]. Since this vicious cycle greatly contributes to increasing neuronal death, we sought to reduce this phenomenon by inhibiting the TRPM7 channel via the administration of carvacrol. Here, we found that seizure-induced ROS production and oxidative injury were decreased by carvacrol administration. Our findings suggest that blocking TRPM7 by carvacrol reduces the accumulation of intracellular free zinc and thereby inhibits neuron death following pilocarpine-induced SE.

Microglia/macrophages are highly flexible glial cell of the CNS, which are recognized to play an important role not only in a healthy CNS but also in various pathological conditions [76]. In general, microglia/macrophages are present in a resting state under physiological conditions and are rapidly activated under pathological conditions, including increased chemokine and cytokine synthesis [77,78,79]. Activated microglia/macrophages gather around degenerating neurons in CNS disease. The first immune defense line, the microglia, responds rapidly to antigens, releasing tumor necrosis factor (TNF)–α, interleukin (IL)-6, and nitric oxide (NO) [80,81]. Thus, microglia/macrophage activation is one of the most common early features of neurological diseases. Microglia/macrophage polarization (divided into M1 and M2) is the mediator of pro-inflammatory and anti-inflammatory responses, respectively [82]. The pro-inflammatory response promotes inflammatory cytokines, which are a type of signaling molecule released from immune cells, such as helper T cells and macrophages, as well as other cell types that promote inflammation [83]. The present study found that the activity of microglia/macrophages that leads to neuronal death following SE involves the activity of M1 microglia/macrophages and carvacrol treatment reduces the activation of M1 microglia/macrophages after SE.

BBB, a unique anatomical and physiological interface between the CNS and peripheral circulation, is essential to provide a suitable environment for neurological function regardless of fluctuations in blood composition [84,85,86]. After epilepsy, pinocytotic activity and tight junction deformation increased and contributed to BBB dysfunction [87,88]. There are various mechanisms through which BBB dysfunction occurs after a seizure: free radical production, cytokine release, glutamate release, and inflammatory cytokines [89,90]. It is well known that BBB permeability increases after a seizure [91,92]. BBB breakdown can be caused by the activation of reactive oxygen species (ROS), matrix metalloproteinases (MMPs), inflammatory cytokines, immune cell extravasation, and leukocyte adhesion [89]. Impaired BBB integrity is mainly indicated by increased barrier permeability. Increased BBB permeability allows several cytokines to enter, such as IL-18 and TNF-α, which are involved in the immune response [93]. In the present study, we showed that carvacrol treatment that has BBB permeability [94,95] reduces seizure-induced BBB disruption by inhibiting the ROS, which increases permeability by destroying the BBB’s integrity.

Oregano has a high content of carvacrol and thymol, which are phenolic antioxidants. Carvacrol is the most effective antifungal and antibacterial agent [96] while thymol is isomeric with carvacrol that strengthen the immune system [97]. However, there are several adverse side effects that can be expected besides weight loss, such as, toxicity due to inhibition of absorption of iron. Others include allergic reactions and die-off effects. If there is massive die-off, one might experience nausea and headache; digestive problems; blood thinning, tingling sensation in the mouth; high blood pressure in some people. In the present animal study, we did not find any of the above behavioral side effects in the carvacrol-treated rats during the entire experiment.

**Figure 8:**
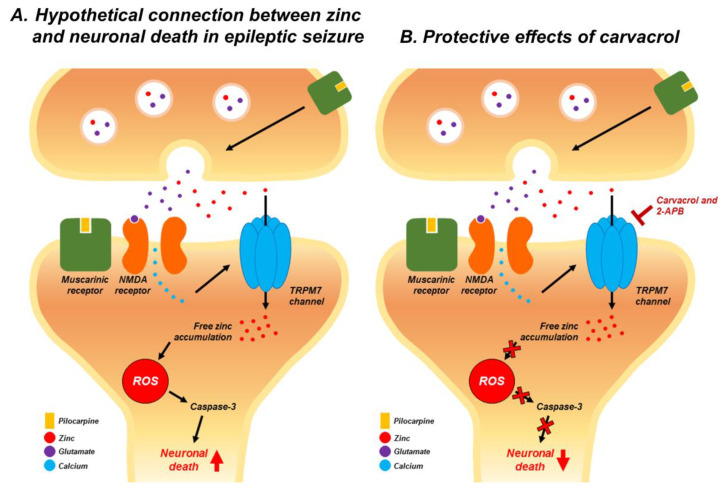
Possible association of zinc with neuronal death after pilocarpine-induced SE. This schematic drawing represents several chain reactions that may occur after carvacrol and 2-APB treatment in pilocarpine-induced SE. (**A**) These are the possible cellular pathways through which neuronal death occurs after pilocarpine-induced SE. (**B**) Blocking TRPM7 by carvacrol and 2-APB can inhibit several chain reactions that are thought to occur following pilocarpine-induced SE.

A previous study demonstrated that the administration of pilocarpine acts on the pre-synaptic and post-synaptic muscarinic receptors, thereby increasing the glutamate levels [98]. This leads to glutamate release from the presynaptic terminals. The released glutamate binds to the postsynaptic N-methyl-D-aspartate receptor (NMDAR) and facilitates calcium influx into the cells [99]. Calcium enters into the cell through NMDAR and then stimulates the TRPM7 channel [100]. Zinc enters through the activated TRPM7 channel and accumulates [26]. Excessive zinc stimulates the ROS production produced by NADPH-oxidase and mitochondria, thereby triggering further intracellular zinc mobilization [101,102]. Thus, excessive zinc and ROS are capable of triggering signaling cascades that ultimately give rise to neuronal death by apoptosis [103,104]. We speculate that carvacrol and 2-APB may prevent initial translocation of zinc into postsynaptic neurons after seizure and then carvacrol reduced over expression of TRPM7 after seizure. The present study found that the inhibition of TRPM7 by carvacrol and 2-APB reduces zinc-mediated apoptotic neuron death following pilocarpine-induced SE (Figure 8).

Limitations of this study are that no highly-specific inhibitors of the TRPM7 channel are currently available, and also a paucity of potential mechanisms to explain the neuroprotective effects of the unspecific inhibitors carvacrol and 2-APB on seizure-induced neuronal death. To further confirm our hypothesis a specific TRPM7 inhibitor is needed for future experiments, since both carvacrol and 2-APB are non-specific inhibitors of TRPM7.

Taken together, our findings indicate that TRPM7 inhibition by carvacrol or 2APB can protect seizure-induced neuronal death via the reduction of intracellular free zinc accumulation. Thus, the present study suggests that TRPM7 channel inhibition may be a potential candidate for preventing epilepsy-induced neuronal death.

## 4. Materials and Methods

### 4.1. Ethics Statement and Experimental Animals

This study was approved in accordance with the Laboratory Animal Guides and Laboratory Animal Rules published by the National Institute of Health. The animal study was conducted in accordance with the experimental animal research project approved under the criteria of the Experimental Animal Research Committee (Protocol # Hallym 2018-29). This study was conducted using 8-week-old male Sprague–Dawley rats (280–350 g, DBL Co, Korea). The animals were adapted for 1 week under a constant temperature (22 ± 2 °C) and humidity (55 ± 5%). Moreover, the room lighting was set to automatically turn on and turn off every 12 h (8:00 and 20:00) to keep it constant. The study was conducted and written according to the Animal Research: Reporting in Vivo Experiments (ARRIVE) guidelines.

### 4.2. Seizure Induction

Seizures were induced using lithium-pilocarpine. Rats were intraperitoneally injected with lithium chloride (LiCl, 127 mg/kg) to boost the action of the muscarinic receptor 19 h before the injection of pilocarpine. Scopolamine (2 mg/kg) was intraperitoneally injected 30 min before pilocarpine injection, which was used to inhibit the peripheral cholinergic properties based on the activity of muscarinic receptors. Pilocarpine (25 mg/kg) was intraperitoneally injected 30 min after scopolamine injection. Status epilepticus (SE) was induced following the injection of pilocarpine [105]. All solutions were dissolved with 0.9% saline and prepared immediately prior to use. SE usually occurs within 20–30 min after pilocarpine injection [106]. The animals were placed one animal per cage to observe their seizure behavior. Seizure behavior was classified according to the process of Racine (facial movements, salivation, hardened hind limbs, forelimb clonus, rearing, and falling) [55]. When Racine stage falling occurs, it is regarded as the onset of seizure. Then, diazepam (10 mg/kg) was intraperitoneally injected 2 h after onset to terminate the seizure induction.

### 4.3. Experimental Design and Inhibitor Treatment

To investigate whether carvacrol reduces neuronal death after seizure, the experimental group was divided into the following periods. In phase 1, carvacrol (Sigma-Aldrich Co., St. Louis, MO, USA) was administered once, and the rats were sacrificed 3 h after seizure. To measure the level of dihydroethidium (dHEt), the animals were divided into 4 groups: (1) sham-vehicle (*n* = 6), (2) sham-carvacrol (*n* = 6), (3) seizure-vehicle (*n* = 6), and (4) seizure-carvacrol (*n* = 5). In phase 2, carvacrol was administered once, and the rats were sacrificed 24 h after seizure. To access the level of zinc translocation, the rats were divided into 4 groups: (1) sham-vehicle (*n* = 6), (2) sham-carvacrol (*n* = 6), (3) seizure-vehicle (*n* = 5), and (4) seizure-carvacrol (*n* = 5). In phase 3, carvacrol was administered once a day for 3 days, and the animal brain was obtained 3 days after seizure. To investigate the effect of carvacrol after seizure, the animals were divided into 4 groups: (1) sham-vehicle (*n* = 5), (2) sham-carvacrol (*n* = 5), (3) seizure-vehicle (*n* = 8), and (4) seizure-carvacrol (*n* = 8). In phase 4, carvacrol was intraperitoneal administered once a day for 7 days, and the experimental animals were sacrificed to obtain brain tissue 7 days after seizure. To evaluate the neuroprotective effect of carvacrol, the experimental animals were divided into 4 groups: (1) sham-vehicle (*n* = 6), (2) sham-carvacrol (*n* = 6), (3) seizure-vehicle (*n* = 10), and (4) seizure-carvacrol (*n* = 10). In phase 5, 2-APB was administered once a day for 3 days, and the brain was obtained at 3 days after seizure. To investigate the effects of 2-APB after seizure, the animals were divided into 4 groups: (1) sham-vehicle (*n* = 5), (2) sham-2PB (*n* = 5), (3) seizure-vehicle (*n* = 5), and (4) seizure-2-APB (*n* = 6).

Carvacrol was dissolved in 0.1% DMSO and intraperitoneally administered at a dose of 50 mg/kg immediately after a 2-h seizure. Control rats were intraperitoneally injected with equal volumes of 0.1% DMSO (diluted 0.9% saline) only (vehicle). The concentration of carvacrol was determined in the previous study [47]. The therapeutic window for carvacrol is calculated as LD50/ED50. LD50 is 1544.5 mg/kg, and ED50 is 35.8 mg/kg, so it is 43.142. Since the dose we used in this experiment is significantly lower than the LD50 concentration. Thus, we believe that the dose we used in this study is sufficiently non-toxic for therapeutic use. In addition, to investigate whether 2-APB reduces neuronal death after seizure, 2-APB was administered once a day for 3 days, and the animal brain was obtained 3 days after seizure. 2-APB was dissolved in 0.9% saline and intraperitoneally administered at a dose of 2 mg/kg immediately after a 2-h seizure. Control rats were intraperitoneally injected with equal volumes of 0.9% saline only (vehicle). The concentration of 2-APB was determined in the previous study [107].

### 4.4. Brain Sample Preparation

Animals were sacrificed at 3 h, 24 h, 3 days, and 7 days after seizure. The animals were anesthetized by an intraperitoneal injection of urethane at a dose of 1.5 g/kg. After anesthesia, the animals were transcardially perfused with 0.9% saline and then with 4% paraformaldehyde. Their brains were quickly removed and post-fixed in 4% paraformaldehyde for one hour. After fixation, the brains were stored in 30% sucrose solution, which served as a cryoprotectant. Two days later, the brain was cut to a thickness of 30 μm using cryostat microtome (CM1850; Leica, Wetzlar, Germany).

### 4.5. Detection of Neuronal Degeneration

Fluoro-Jade B (FJB) staining was performed to evaluate the effect of carvacrol on neuronal degeneration after seizure. FJB staining was carried out as defined by Schmued et al. [59,108]. After cryosection, the tissue cut to a thickness of 30 μm was placed on a slide coated with gelatin. Then, the slide was immersed in 100% and 70% alcohol for 3 and 1 min, respectively. The slide was next washed with DW for 1 min and immersed in 0.06% potassium permanganate for 15 min. Then, it was washed for 1 min with DW and immersed in 0.001% Fluoro-Jade B solution (Histo-Chem Inc., Jefferson, AR, USA) for 30 min and next washed with DW, dried in an oven for at least 30 min, and the slide was covered with DPX (Sigma-Aldrich Co., St. Louis, MO, USA). The stained tissue was observed at 450–490 nm using an Axioscope microscope (Carl Zeiss, Munchen Hallbergmoos, Germany). Every 6 th section was selected from bregma to caudal from 2.92 to 4.56 nm. The quantification of FJB-positive neurons was conducted by a blind observer.

### 4.6. Detection of Zinc Translocation

6-Methoxy-(8-p-toluene sulfonamido) quinolone (TSQ) staining was performed to evaluate the effect of carvacrol on zinc translocation after seizure. After 24 h of administration of carvacrol, the rat was anesthetized to obtain brain tissue, and the obtained brain tissue was frozen in dry ice for 1 min. After that, cryosection was carried out with a thickness of 10 μm. The cut slices were stuck to the coated slides and stained with 0.001% TSQ solution (Molecular Probes, Eugene, OR, United States) for 1 min. Then, the slides were washed with 0.9% saline for 1 min and observed with an Axioscope microscope (Carl Zeiss, Munchen Hallbergmoos, Germany). Quantification of the TSQ positive cells was conducted by a blind observer according to the previously described method [109].

### 4.7. Detection of Reactive Oxygen Species (ROS) Activation

Dihydroethidium (dHEt) staining was performed to evaluate the effects of carvacrol on reactive oxygen species (ROS) after seizure. When rats were injected with pilocarpine, dHEt was also injected (5 mg/kg, life technologies, Carlsbad, CA, USA). At 3 h after seizure induction, we obtained brain tissue. The obtained brain tissue was frozen after being sectioned with a thickness of 30 μm. Then, the tissue was washed three times in phosphate-buffered saline (PBS) for 10 min, placed on a coated slide, and mounted with DPX solution. The stained tissue was observed at wavelength of 518 nm/605 nm using an Axioscope microscope (Carl Zeiss, Munchen Hallbergmoos, Germany). The ethidium signal intensity was quantified by obtaining 5 coronal sections (2.92 to 4.56 nm from bregma to caudal). Et fluorescence intensity was measured by ImageJ (National Institutes of Health, Bethesda, MD) and expressed as mean gray value.

### 4.8. Immunofluorescence Assay

To evaluate the effect of carvacrol treatment after seizure, we performed an immunofluorescence assay. Brain tissue was obtained 3 days after seizure. The obtained brain tissue was frozen after being sectioned to a thickness of 30 μm. Then, the tissue was washed three times in PBS for 10 min. Next, pretreatment was performed with a solution containing 90% methanol, distilled water, and 30% hydrogen peroxide to completely remove blood from the blood vessels in the tissues. The brain tissue was then immersed in an antibody solution in PBS containing 0.3% Triton X-100 and kept overnight at 4 °C. The primary antibodies used in this study were as follows: rabbit anti-TRPM7 (diluted 1:400; Alomone labs, Jerusalem, Israel), mouse andti-4HNE (diluted 1:500; Alpha Diagnostic Intl. Inc., San Antonio, TX, USA), goat anti-Iba1 (diluted 1:500; Abcam, Cambridge, UK), mouse anti-CD68 (diluted 1:100; Bio-Rad, Hercules, CA, USA), rabbit anti-GFAP (diluted 1:1k; Abcam, Cambridge, UK), mouse anti-SMI71 (diluted 1:500; Covance, Princeton, NJ, USA), rabbit anti-cleaved caspase3 (diluted 1:250; Cell signaling, Danvers, MA, USA), and mouse anti-NeuN (diluted 1:500; Millipore, Billerica, MA, USA). Next, the tissue was washed three times in PBS for 10 min and immersed with the secondary antibody in PBS containing 0.3% Triton X-100 for 2 h. A secondary antibody with fluorescence for each primary antibody (TRPM7, 4HNE, Iba1, CD68, GFAP, SMI71, and cleaved caspase 3) was used (diluted 1:250; Invitrogen, Grand Island, NY, USA). We also counterstained with DAPI (4,6-diamidino-2-phenylindole; diluted 1:1k; Invitrogen, Grand Island, NY, USA). Then, the fluorescence stained sections were placed on a coated slide and mounted with a DPX solution (Sigma-Aldrich). Quantification used the Image J software (National Institutes of Health, Bethesda, MD). This quantification was performed using 5 coronal sections from 2.92 to 4.56 nm from bregma to caudal. This was quantified using a method modified from the previously described method [110].

### 4.9. Immunohistochemistry Assay

To evaluate the effect of carvacrol treatment after seizure, we performed an immunohistochemistry assay. The brain tissue prepared as above was immersed in a solution of mouse anti-NeuN antibodies (diluted 1:500, Millipore, Billerica, MA, USA) in PBS containing 0.3% Triton X-100 and kept overnight at 4 °C. Then, the brain tissue was immersed in a solution of anti-mouse IgG (diluted 1:250; Vector, Burlingame, CA, USA) in PBS containing 0.3% Triton X-100 and treated for 2 h. To analyze endogenous IgG leakage after seizure, sections were incubated in the anti-rat IgG (diluted 1:250; vector, Burlingame, CA, USA) at room temperature for 2 h. Then, the ABC complex solution (Vector, Burlingame, CA, USA) was treated at room temperature for 2 h. The tissue was colored for 1 min 30 s using 3,3’-diaminobenzidine (DAB ager, Sigma-Aldrich Co., St. Louis, Mo, USA) dissolved in a 0.01 M PBS buffer. Then, the stained tissue was placed on a coated slide and mounted in a Canada balsam (Junsei chemical, Chuo-ku, Tokyo, Japan) solution. The quantification used the Image J software (National Institutes of Health, Bethesda, MD) to measure the IgG intensity and NeuN positive cells. The analysis was performed from bregma to caudal with coronal sections obtained from 2.92 to 4.56 nm.

### 4.10. Western Blot

Hippocampal tissues were homogenized with lysis buffer containing RIPA buffer (Cat.IBS-BR002, iNtRON, Seongnam, Republic of Korea), protease inhibitor (Cat.11697498001, Sigma, St. Louis, MO, USA) and phosphatase inhibitor (Cat.4906845001, Sigma, St. Louis, MO, USA). The tissue was incubated on ice for 30 min and centrifuged at 14,000 rpm for 20 min at 4 ℃. Then, only the supernatant was obtained, and protein quantification was carried out using a Bradford protein assay. The quantified protein was prepared at a concentration of 25 μg and electrophoresed on SDS-PAGE gel. Then, the separated proteins were transferred to PVDF membrane. The membrane was blocked by incubating 5% skim milk and 3% BSA at room temperature to prevent non-specific staining. The membrane was incubated with primary antibody overnight at 4 ℃. We used the following primary antibodies: TRPM7 (diluted 1:200; Alomone labs), and β-actin (diluted 1:10,000; Cell signaling). The membrane was then washed with TBS-T (Cat.190-6435, Bio-Rad, Hercules, CA, USA) three times for 10 min. The membrane was incubated for 1 h at room temperature using anti-rabbit IgG and anti-mouse IgG secondary antibody conjugated with horseradish peroxidase (HRP) (diluted 1:5000; Ab frontier). The protein on the PVDF membrane was detected using chemiluminescence bioimaging instrument (Amersham imager 680, Marlborough, USA). The intensities of bands were measured by densitometry using a scanner with Image J (National Institute of Health, Bethesda, MD, USA) and quantitative analysis was peformed.

### 4.11. Data Analysis

In this study, a program called image J (National Institute of Health, Bethesda, MD, USA) was used for data analysis, and the mean gray value or % area was measured according to the experiment to be analyzed. In order to analyze the number of neurons, the blind observer excluding the experimenter directly counted the number of neurons through the blind test. All data were expressed as the mean ± SEM. Comparisons between the vehicle- and carvacrol-treated groups were performed with a two-tailed unpaired Student’s t-test and a Mann–Whitney U test. To compare the values among the four groups, the remaining data were analyzed by a Kruskal–Wallis test with a post-hoc analysis using Bonferroni correction. *p*-values below 0.05 were considered statistically significant.
